# Liver Transplantation for Unresectable Calcifying Nested Stromal Epithelial Tumor: Case Report With a 1-Year Follow-Up and Review of Literature

**DOI:** 10.3389/fsurg.2022.875782

**Published:** 2022-05-02

**Authors:** Emilia Kruk, Konrad Kobryń, Paweł Rykowski, Benedykt Szczepankiewicz, Waldemar Patkowski, Krzysztof Zieniewicz

**Affiliations:** ^1^Department of General, Transplant and Liver Surgery, Medical University of Warsaw, Warsaw, Poland; ^2^Department of Pathomorphology, Medical University of Warsaw, Warsaw, Poland

**Keywords:** CNSET, liver transplantation, liver resection, liver tumor, liver oncology

## Abstract

**Introduction:**

Calcifying nested stromal epithelial tumor (CNSET) is an extremely rare diagnosis among patients treated for primary hepatic neoplasms. There are only 45 cases reported worldwide. Histopathological characteristics are well-demarcated nests of spindle and epithelioid cells in a dense desmoplastic stroma with variable calcification and ossification. It is mostly diagnosed in children and young females. Treatment strategies implemented for the management of CNSET include radiofrequency ablation, transarterial chemoembolization, surgical resection, adjuvant and neoadjuvant chemotherapy, and liver transplantation. Given the small number of available cases, there are still no established standards of treatment for this neoplasm.

**Case Presentation:**

A 28-year-old female diagnosed with CNSET presented mild abdominal pain, with normal laboratory values. The tumor was initially deemed unresectable, therefore, the patient was disqualified from liver resection. Further deterioration of the patient's clinical condition and local tumor progression led to qualification for liver transplantation. The patient underwent liver transplantation 1 year following initial diagnosis and a 12 months recurrence-free period was observed. During the course of treatment, she did not receive systemic chemotherapy, radiotherapy, or loco-regional treatment.

**Conclusion:**

Multiple strategies have been implemented for the treatment of CNSET, with liver resection providing the best outcomes. Transarterial chemoembolization, radiofrequency ablation, and radiotherapy are reported to be insufficient in the management of this tumor. Various chemotherapy regimens turned out to be ineffective as well. There have been only eight reported cases of patients undergoing liver transplantation for CNSET, with tumor recurrence in two cases. CNSET appears to be a neoplasm with low malignancy potential, although an aggressive progression has subsequently been reported. Further investigation is still required in this field.

## Introduction

Calcifying nested stromal epithelial tumor (CNSET) is an extremely rare diagnosis among patients treated for primary hepatic neoplasms. There are only 45 cases reported worldwide (1–29). CNSET was first described in 2001 by Ishak et al. (30) and since then has been appearing in the literature under various names e.g., desmoplastic nested spindle cell tumor, ossifying stromal-epithelial tumor, malignant mixed epithelial, and stromal tumor (27). The origin of this neoplasm is still unknown. One of the hypotheses is that it's “a derivative of hepatic mesenchymal precursor cells, with a possible differentiation along the bile duct lineage” (24). CNSET histopathological characteristics are well-demarcated nests of spindle and epithelioid cells in a dense desmoplastic stroma with variable calcification and ossification (27). It is mostly diagnosed in young females with the youngest reported patient being two (9) and the oldest being 34 years old (27). The tumor usually does not manifest early symptoms and is mostly diagnosed incidentally, as a singular mass averaging 12.6 cm in size (30). Laboratory results lack abnormalities, although some authors (2, 4, 7, 11, 12, 21, 25, 28) reported the coexistence of CNSET with Cushing's Syndrome, therefore, elevated levels of adrenocorticotropic hormone (ACTH) may be observed.

Multiple treatment strategies have been implemented for the management of CNSET including radiofrequency ablation, surgical resection, adjuvant, and neo-adjuvant chemotherapy, and liver transplantation. Surgery is the most successful method, provides the best outcomes for treatment. There were only four reported cases of recurrence among 35 patients that underwent liver resection as the sole surgical intervention (7, 8, 16, 26). To the best of our knowledge, only six patients underwent liver transplantation (LT) for primarily unresectable CNSET (1, 3, 11, 12, 24, 25), and two patients were transplanted due to recurrence (2, 10). Due to the low occurence of this tumor, data in the literature is still limited. There are plenty of questions left unanswered, however, the underlying issue concerns whether LT is supreme to liver resection as a method of treatment.

## Case Presentation

On October 2019, a 28-year-old female patient was referred to the Department of General, Transplant and Liver Surgery of the Medical University of Warsaw due to an incidental tumor finding in the right liver. At admission, laboratory test results were found to be normal. She presented mild discomfort in the upper-right quadrant of the abdomen throughout the previous 6 weeks. A core needle biopsy was performed. Pathologists diagnosed an intrahepatic cholangiocarcinoma tumor. Due to inadequate future liver remnant (tumor size of 200 × 180 × 150 mm with right hepatic vein involvement) the tumor was claimed unresectable at that stage. The patient was referred to the oncologist for systemic therapy. A second core needle biopsy was performed, but this time a different tumor was diagnosed (CNSET).

Given an alternative diagnosis, the patient did not receive chemotherapy and was re-evaluated for surgical treatment. Multiple imaging was performed which presented local progression of the disease, increase in tumor size, diaphragmatic infiltration, right and middle hepatic vein obstruction, and right portal vein obstruction. Due to this tumor progression, she was once again disqualified from resection.

**Figure 1:**
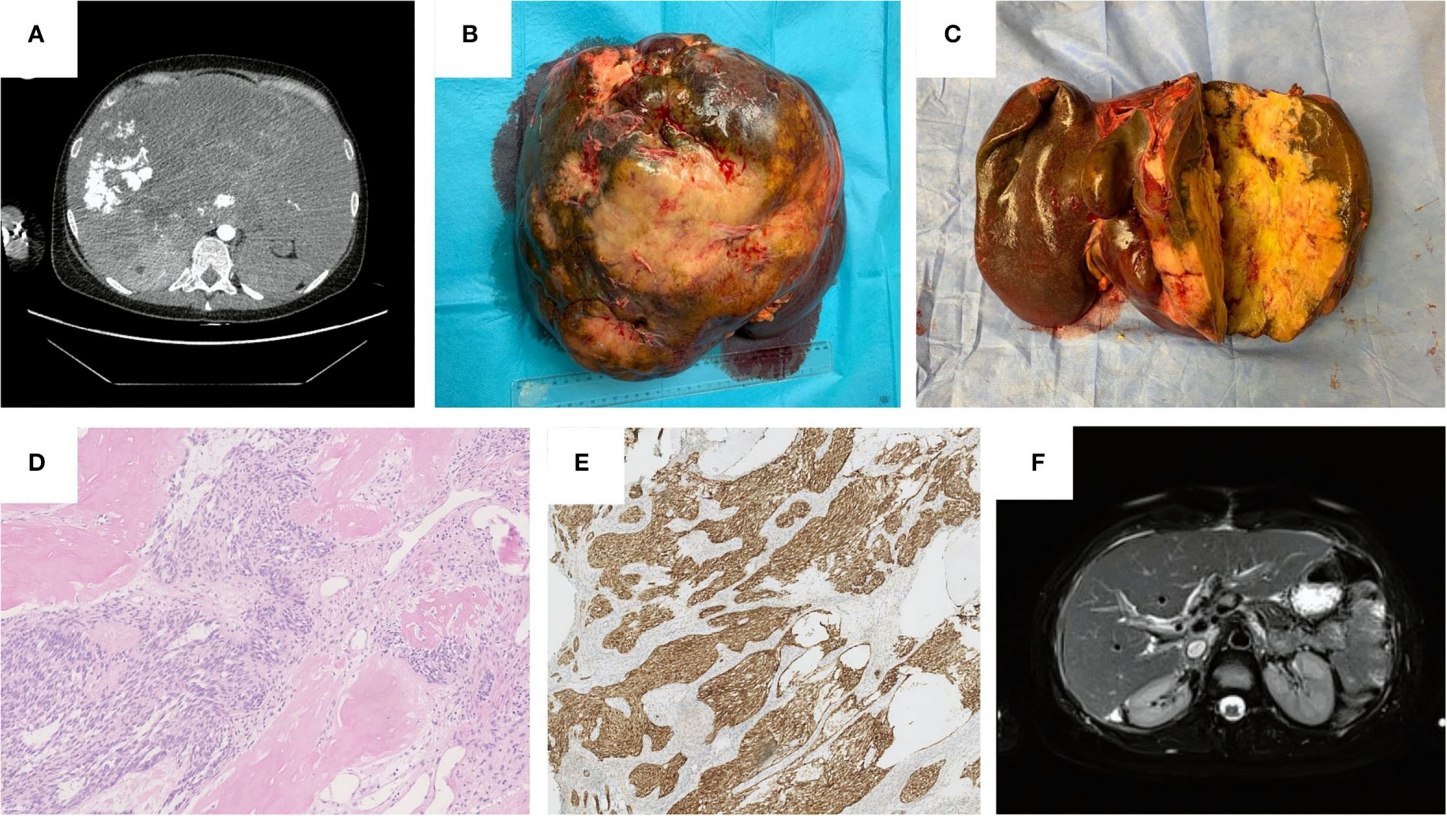
**(A)** Computed Tomography Scan before liver transplantation, November 2020. **(B)** Gross appearance of the tumor in explanted liver. **(C)** Cross-section of the tumor in explanted liver. **(D)** Histopathological features: Eosin-haematoxylin dye. Well-defined nests composed of short fascicles of bland looking spindle cells surrounded by desmoplastic stroma and areas of ossification, neocholangioli inside desmoplastic stroma are visible. **(E)** Histopathological features: nuclear and cytoplasmic immunohistochemical reaction with beta-katenin. **(F)** Magnetic Resonance Imaging 8-months after liver transplantation, July 2021.

Throughout the course of consecutive months, the patient's quality of life suffered following increasing abdominal pain, uncontrollable hiccups, slight peripheral edema, and the emergence of paraneoplastic erythematous skin lesions. Yet, she did not receive systemic treatment. In August 2020, a control Computed Tomography Scan revealed tumor volume growth occupying the right liver along with segment IV and segment I with the obstruction of intrahepatic inferior vena cava (Figure 1A). Positron Emission Tomography (PET) gave no evidence of metastases. Due to symptomatic tumor progression, no evident signs of extrahepatic disease, and no other surgical treatment options available, the patient was approved for liver transplantation. Preceding LT, her liver enzymes were elevated (alanine transaminase 331 IU/ml, aspartate transaminase 1,615 IU/ml), with total serum bilirubin 7.54 mg/dl, gamma-glutamyl transpeptidase 65 U/L. Tumor markers remained negative.

The patient underwent LT on 19 November, 2020, with the use of veno-venous bypass and end-to-end cavo-caval anastomosis. During hepatectomy, a 65 × 30 × 10 mm part of the diaphragm was excised en-bloc with the liver due to tumor infiltration, excised specimen weighed 4,800 g. A standard immunosuppression regimen of tacrolimus, prednisone, and mycophenolate mofetil was maintained.

Histopathological examination presented a well-circumcised tan-white tumor of a maximum size of 26 cm and another 6 mm infracapsular tumor (Figures 1B,C). Microscopic examination revealed typical morphological features of CNSET. The tumor was composed of well-defined nests surrounded by desmoplastic stroma and areas of ossification. These nests were composed of short fascicles of bland looking spindle cells and inside the desmoplastic stroma neocholangioli were observed (Figure 1D). A characteristic feature of this tumor is the nuclear and cytoplasmic immunohistochemical reaction with beta-katenin (Figure 1E), which corresponds with previously reported histopathological characteristics of CNSET (27). In our case, two additional tissue structures were found: 1st—spindle-cell high-grade sarcoma, 2nd—primitive high-grade plasmacytoid sarcoma. The first tissue architecture was found in a 6 mm satellite tumor, which, therefore, should be treated as an intrahepatic metastasis, these cells were also found forming an embolus in one of the segmental hepatic veins. As far as we know, the histological findings that we described in this study have not been previously reported in patients diagnosed with CNSET.

The patient experienced a 10-min asystolic cardiac arrest on a post-operative day (POD) 6, most likely attributed to sudden hypoxemia caused by an iatrogenic diaphragmatic injury that subsequently led to respiratory failure with the need for mechanical ventilation and sedation until POD 23. The prolonged intubation led to a Serratia Marcescens infection confirmed in blood and bronchoalveolar fluid cultures, infection was treated according to antibiogram with a good outcome.

The patient was discharged on POD 42 in good general condition with normalized laboratory liver parameters.

The last outpatient follow-up was in January 2022 (Table 1), where liver enzymes remained normal and apart from Ca125 (210 U/ml), tumor markers remained normal. Multiple diagnostic imaging carried out throughout the year showed no signs of recurrence (Figure 1F).

**Table 1:** Timeline.

**Date**	**Events**
September 2019	- Onset of symptoms: mild pain in upper right quadrant of the abdomen
October 2019	- Admission to the Department of General, Transplant and Liver Surgery of the Medical University of Warsaw - Radiological imaging: tumor measuring 17 × 17 × 18 cm - Disqualification from surgical intervention - First core needle biopsy: diagnosis of intrahepatic cholangiocarcinoma
November 2019–February 2020	- Second core needle biopsy: diagnosis of CNSET - Disqualification from systemic chemotherapy
March 2020	- Readmission to the Department of General, Transplant and Liver Surgery of the Medical University of Warsaw - CT Scan: tumor measuring 18 × 16 × 22 cm - Disqualification from surgical intervention
April–October 2020	- Further worsening of symptoms: constant abdominal pain, uncontrollable hiccups
November 2020	- CT Scan: tumor measuring 18 × 20 × 26 cm right and middle hepatic vein and intrahepatic inferior vena cava obstruction - Patient approved for liver transplantation
November 20, 2020	- Orthotopic liver transplantation
December 31, 2020	- Discharge from the hospital
January 2022	- Latest follow-up

Stenosis of the upper cavo-caval anastomosis with the reversed flow in renal veins was identified and managed with a series of percutaneous transluminal angioplasty using EverCross and Atlas Gold balloon catheters with an adequate outcome. The patient throughout the entire course of treatment did neither receive chemotherapy nor radiotherapy.

## Discussion

We presented a case of a patient with unresectable CNSET, an extremely rare primary hepatic tumor. Our patient being a young female, having little to no symptoms at the initial presentation was consistent with previously reported cases (19, 31). One of the hypotheses is that CNSET might develop from benign calcified hepatic lesions. There are reports in the literature of four patients with a history of calcifying liver nodules that were believed to be benign haemangiomas, and that later developed CNSET (12). However, in our case, we had no evidence of previously identified hepatic nodules. Although some authors report ectopic ACTH production resulting in the development of a paraneoplastic Cushing's syndrome that resolves after tumor excision (2, 4, 7, 11, 12, 21, 25, 28), we did not find any symptoms of Cushing's syndrome in our patient.

There are reports of patients with Beckwith–Wiedemann syndrome (BWS) that developed CNSET (11, 24). BWS is a congenital overgrowth syndrome that is characterized by an increased risk of carcinogenesis, specifically embryonal tumors, in children (11). The number of available cases regarding CNSET in patients with BWS is scarce, thus cannot stand to prove a correlation between the two clinical entities. Given the suspected origin of CNSET as being a derivative of progenitor cells with the potential for multi-phenotype differentiation or hepatic mesenchymal precursor cells, with differentiation possibilities along the bile duct lineage, it seems worth investigating whether patients with the congenital predisposition of embryonal tumors are more prone to developing this rare liver tumor (12, 24).

All available reports across Pubmed and Cochrane library regarding CNSET describe it as a primary hepatic tumor. There are no reports of CNSET developing primary lesions in other organs. There are however two cases describing distant metastases to the lungs and bone (10). We still know very little of the biology of this tumor due to very few cases reported worldwide and a lack of follow up.

Considering the limited number of reports in the literature, therapeutic options for CNSET rely mostly on surgical resection. Liver resection has proven positive outcomes with reported recurrence in just 4/35 cases of patients for whom liver resections were the only surgical intervention. There have been two cases of liver and lymph node recurrence (16, 26) and two cases of local hepatic recurrence (7, 8). There were no distant metastases reported in that group of patients.

In all those cases there were no signs of metastases in preoperative imaging, surgeons obtained R0 resections, and none of the patients received chemotherapy prior to surgery.

The most common treatment for recurrence has been systemic chemotherapy. Interestingly the hepatocellular carcinoma regimen turned out to be unsuccessful (26), also the high-risk soft tissue sarcoma protocol only led to minimal tumor shrinkage with suspected cavitation (7), cisplatin/isophosphamide 4-course regimen implemented by Melatani et al. (16) was unsuccessful as well. This leaves us with the unsatisfactory feeling that we do not yet have a successful drug therapy for this tumor.

Radiofrequency ablation (RFA) sparked hope but soon proved far from optimal. Two patients were reported to have been treated with RFA (8, 12, 26). The only successful case was reported by Heywood et al. (8) and Makhlouf et al. (12) where RFA was carried out in the management of recurring CNSET, 6 years after patients with initial hepatectomy developed two new foci, underwent radiotherapy, and remained well after 14 years of follow up.

Meletani et al. (16) and Tsuruta et al. (26) reported two cases of patients that underwent transcatheter arterial chemoembolization (TACE) for local recurrence of CNSET. Results were a progression of the disease in both cases. Clearly, this treatment is not the method of choice for CNSETs.

Adjuvant and neoadjuvant systemic chemotherapy have been applied for the treatment of CNSET utilizing regimens that are commonly used for hepatoblastoma or sarcoma (25), but those therapeutic strategies showed little to no effect on decreasing the tumor size or inducing histological response (32).

To the best of our knowledge, there were eight cases of patients diagnosed with CNSET that underwent liver transplantation. Six of them as a primary method of treatment (1, 3, 11, 12, 24, 25), and two underwent transplantation for recurrence (2, 10) (Table 2).

**Table 2:** Patients undergoing liver transplantation for CNSET.

**References**	**Age**	**Sex**	**Symptoms**	**Max tumor size (cm)**	**Chemotherapy**	**Other treatment**	**Follow-Up**
Brodsky et al. (2)	17.5	Female	Abdominal pain, cushingoid features	22 cm	None	Partial hepatectomy 12 months prior to LTx	No follow-up after LTx
Hommann et al. (10)	16	Female	none	30 cm	High-Risk soft tissue sarcoma protocol + Imatinib for recurrence, no response	Left bisegmentectomy 25 months prior to LTx, TACE for recurrence	Recurrence 28 months post-LTx with lung metastases, deceased 37 months post-LTx
Makhlouf et al. (12)	18	Female	None	20 cm	None	None	Deceased 40 months after LTx of postoperative complications, no recurrence
Assmann et al. (1)	16	Male	None	27 cm	Neoadjuvant CHT (cis-platinum, doxorubicine, isofosfamide), no response	None	No recurrence after 24 months
Schaffer et al. (24)	14	Female	Abdominal distention, Cushing's syndrome	12 cm	2-cycles of neo-adjuvant chemotherapy, no response	None	No recurrence after 10 months
Khoshnam et al. (11)	14	Female	Abdominal pain and swelling, Cushingoid features	12 cm	Neoadjuvant CHT, no response	None	Alive, no recurrence
Tehseen et al. (25)	13	Female	Abdominal pain, Cushingoid features	17.3 cm	Neoadjuvant CHT (vincristine, cyclophosphamide, actinomycin), no response	None	No recurrence after 28 months
Garg et al. (3)	8	Female	Pancytopenia	–	CHT for recurrence, on PET/CT the aortocaval LNs became fluorodeoxyglucose uptake negative	Proton beam radiation therapy for bone metastases	Recurrence 2 months post-LTx, bone and lymph node metastases

In the available reports that provide reasons for qualifying CNSET patients for LT, among the most common indications is the exhaustion of possibilities for radical surgical resection, whether it be tumor size, vascular involvement, significant mass effect, or risk of progressive liver failure (1, 3, 10, 11, 24, 25). In particular cases, patients were qualified for LT due to poor response to systemic treatment (1, 11, 24).

Poland, like many other countries, has experienced a significant decline in the necessity of transplanting cirrhotic hepatitis C-virus (HCV) positive patients. This is mainly due to the fact, that for several years now, direct-acting antiviral agents have been available and successfully administered to this group of patients. This led to a definitive decrease in HCV-positive patients put on the liver transplant waiting list, which in turn has made substantial eligibility for treatment of other hepatic disorders, especially liver tumors. Our decision-making for transplant qualification in cases such as this one is multifactorial. It is based on our center's experience and a near total of 3,000 liver transplants performed throughout the last three decades; evidence in the literature of successful liver transplantation without tumor recurrence, as observed in cholangiocarcinoma patients; the above-mentioned transplant waiting list optimization through lesser numbers of qualified HCV (+) patients. Reasons specifically concerning this patient that led to this transplantation were dictated primarily due to the lack of other therapeutic options in a young, previously healthy individual and progressive but slow tumor growth throughout the 2 years preceding LT. Liver resection was not possible due to the involvement of hepatic veins, and insufficient estimated future liver remanent. Furthermore, quality of life was severely impaired namely by, tumor mass restricting the flow inside the inferior vena cava (IVC) causing systemic circulation congestion or the so called IVC syndrome; diaphragmatic infiltration causing abdominal pain requiring strong opioid administration. The extrahepatic disease was ruled out in consecutive radiology examinations. At LT qualification her patient's model for end stage liver disease score equaled 23 points. The symptoms were clearly associated with the mass effect. It became understandable that taking out the tumor would free the patient from her symptoms, but by being deprived of the possibility to perform a safe and successful liver resection, transplantation became the only resort. Additionally, histopathological examination ruled out the diagnosis of a more aggressive cholangiocarcinoma, which justified the use of a liver graft for this case. Taking all of the above into account, a transplant multidisciplinary team including oncologists, unanimously qualified this patient for deceased donor LT.

In the group of 6 transplanted patients, one had documented recurrence. An 8-year-old female that relapsed 2 months post-transplantation with bone metastases was treated with proton beam radiation therapy for bone lesions and systemic chemotherapy for aorto-caval lymph node metastases. We did not find data on the applied regimen, but the authors report negative uptake of fluorodeoxyglucose by the lymph nodes in PET/CT after chemotherapy administration. It is worth noticing that this patient had suspected lymph node metastases in PET/CT prior to LT. The authors do not provide post-transplant follow-up of this patient (3). Four patients received neoadjuvant chemotherapy. In two cases information about the applied regimen was obtainable. Authors report using vincristine/cyclophosphamide/actinomycin and cis-platin/doxorubicin/isophosphamide regimens but without response (1, 25). In the other two cases, there is no data about regimens applied, although we know that no response was achieved by either (11, 24). There was no reported tumor recurrence in this group.

Two patients that underwent transplantation as a second-line therapy both had been diagnosed with recurrent disease after undergoing first-line surgical treatment. The first patient was a 16-year-old girl treated with liver resection, in follow-up she has been diagnosed with numerous new liver lesions that had been treated with TACE, systemic chemotherapy (high-risk soft tissue sarcoma protocol + Imatinib) with no response and was subsequently transplanted. She recurred at 28 months post-transplantation, manifesting bilateral lung metastases. Further treatment was discontinued, and the patient died 37 months post LT (10). In another case where a 17, 5-year-old female had a primary tumor resected with negative histopathological margins and did not receive chemotherapy. She recurred 12 months following the liver resection and underwent an LT. Follow-up information has not been reported in this case (2).

Most authors reporting the cases of patients receiving LT for CNSET do not describe exact immunosuppression regimens, so we remain unaware of potential modifications. Only three authors provided information about used drugs. Hommann et al. implemented Basiliximab, steroids, and Tacrolimus that was subsequently augmented with Mycophenolate Mofetil, on POD 10 patients who experienced mild rejection treated with low dose steroids (10). Following the diagnosis of recurrence, it was switched to Sirolimus.

Tehseen et al. report using Basiliximab, steroids, and Tacrolimus, while Gard et al. reports using Tacrolimus alone, we do not know whether in this case immunosuppression was altered after recurrence (3, 25).

Our patient was started on standard immunosuppression used at our facility that included induction with 250 mg Methylprednisolone, later switched to 20 mg Prednisone, and subsequently started on 2 mg Tacrolimus and 250 mg Mycophenolate Mofetil. Her current regimen as of March 2022 consists of 3 mg Tacrolimus and 2 mg Everolimus daily. We did not observe graft rejection, adverse events, or recurrence during hospitalization until this day. Although this was not a hepatocellular carcinoma (HCC), CNSET is currently recognized as a primary liver tumor. Nowadays, we know that the use of mammalian target of rapamycine (M-TOR) inhibitors in HCC LT recipients reduces the recurrence rate and we stand in favor of similar immunosuppression standards toward encounters of rare hepatic tumors. From our own observations, we do not see the need for reduced, but possibly low levels of calcineurin inhibitors (CNIs) in the first weeks post-liver transplantation. As our experience is limited in the matter of CNSET we wish to believe that low dose CNIs with mycophenolate mofetil and M-TORs will be acceptable in prolonging graft survival and the recurrence-free period.

CNSET appears to be a neoplasm with low malignancy potential, although some authors report aggressive disease progression with local recurrence (2, 7–9, 12, 16, 26). Only two authors describe distant metastatic CNSET, both in patients that received LT and received immunosuppression. Furthermore, in one patient pre-operative imaging implied metastatic lymph nodes (3, 10).

Our experience is limited to the presented case of our female patient. She had a high chance of an unfavorable course of the disease given that two additional tissue structures suspected of higher malignancy potential and intrahepatic metastasis were found during pathomorphological examination. Although liver resection remains the most feasible and successful treatment, we believe that in cases such as ours, where large tumor volume is present along with malignant pathology examination, the best therapeutical option is liver transplantation. Immunosuppression does not require alteration and a 1-year observation period does not implicate early recurrence. With the awareness that to this day we do not have a chemotherapy protocol effective enough to prevent the recurrence of CNSET, a safe and margin-free liver resection is the “golden choice” of treatment. LT would then be advocated only in cases where a safe liver resection may be jeopardized.

Given the extremely small group of patients with CNSET worldwide, that underwent LT, a closer look at this disease is mandatory. Reporting of long-term follow-up for these patients is in serious need and would be much appreciated.

## Patient's Statement

We contacted the presented patient on January 2022 to inquire about her views and insights on implemented treatment. The patient stated that throughout the course of treatment she has been overall satisfied with the doctor's approach to finding the most optimal solution for her case. During the diagnostic process, she was tormented by uncertainty, since she was aware of the rarity of her condition, she admitted that she tried to research the topic of CNEST on her own but was unable to find any accessible information dedicated to patients. On being asked for the biggest struggle she had to overcome before liver transplantation, she stated that the biggest obstacle was abdominal pain that worsened in a few months preceding liver transplantation and prevented the patient from working, disabled her from performing everyday tasks, and demanded intake of opioid pain medications that heavily altered her ability to focus on basic activities. Asked how her life has been since transplantation she said that she's been working full-time job, is independent, complies with the ordered immunosuppression regimen, and is regularly controlled in the outpatient clinic. She declared that liver transplantation saved her life and wanted to emphasize that she is very much grateful that she has been able to receive successful treatment for her condition. She stated that she is glad that her case will be submitted for publication and believes that it can be helpful in finding treatment solutions for patients with the same diagnosis.

## Data Availability Statement

The original contributions presented in the study are included in the article/supplementary material, further inquiries can be directed to the corresponding author/s.

## Ethics Statement

Ethical review and approval was not required for the study on human participants in accordance with the local legislation and institutional requirements. The patients/participants provided their written informed consent to participate in this study.

## Author Contributions

EK and KK contributed to the manuscript by writing draft manuscript, writing the discussion and the final manuscript, and literature review. PR, BS, WP, and KZ contributed to manuscript revision, read, and approved the submitted version. All authors contributed to the article and approved the submitted version.

## Conflict of Interest

The authors declare that the research was conducted in the absence of any commercial or financial relationships that could be construed as a potential conflict of interest.

## Publisher's Note

All claims expressed in this article are solely those of the authors and do not necessarily represent those of their affiliated organizations, or those of the publisher, the editors and the reviewers. Any product that may be evaluated in this article, or claim that may be made by its manufacturer, is not guaranteed or endorsed by the publisher.
